# Severe Hyponatremia Following Bilateral Pleural Catheter Placement in a Patient With Persistent Chylous Effusions

**DOI:** 10.7759/cureus.92542

**Published:** 2025-09-17

**Authors:** Joshua Boster, Michael Goertzen, Horiana Grosu

**Affiliations:** 1 Interventional Pulmonology and Critical Care, MD Anderson Cancer Center, Houston, USA; 2 Pulmonary and Critical Care Medicine, MD Anderson Cancer Center, Houston, USA; 3 Pulmonary Medicine, MD Anderson Cancer Center, Houston, USA

**Keywords:** chylous, hyponatremia, indwelling pleural catheter, lymphoma, pleural effusion

## Abstract

Severe hyponatremia following indwelling pleural catheter (IPC) placement in patients with high-output chylous pleural effusions is a rare and infrequently reported complication. We report a case of a 52-year-old male with relapsed/refractory follicular lymphoma who developed severe hypo-osmolar hyponatremia one month after bilateral IPC placement for symptomatic chylous pleural effusions. High-volume drainage led to significant sodium loss, necessitating intensive care unit admission. Management with hypertonic saline, free water restriction, oral urea, and restricted IPC drainage, followed by thoracic duct embolization, improved serum sodium levels. This case highlights severe hyponatremia as a life-threatening consequence of high-output chylous effusions post IPC placement, emphasizing the need for careful electrolyte monitoring and restricted drainage to prevent severe electrolyte imbalances.

## Introduction

Chyle is a milky-white fluid that is composed primarily of lymph and emulsified dietary fats that are absorbed from the small intestine during digestion. Once absorbed, chyle travels through the mesenteric lymphatic vessels into the cisterna chyli and then ascends via the thoracic duct to drain into the venous circulation at the junction of the left subclavian and internal jugular veins. Disruption along this pathway can result in chyle leakage into body cavities such as the pleural or peritoneal space, resulting in chylothorax or chylous ascites. Chyle closely resembles the electrolyte composition of plasma and contains substantial amounts of protein and fat [1,2]. Drainage of approximately 2 liters of chyle daily, such as in this patient, equates to a daily sodium deficit of 260-280 mmol. In cancer patients, this sodium loss can be compounded by decreased oral intake due to nausea and vomiting, which may result in a progressively negative sodium balance over time.

## Case presentation

**Figure 1:**
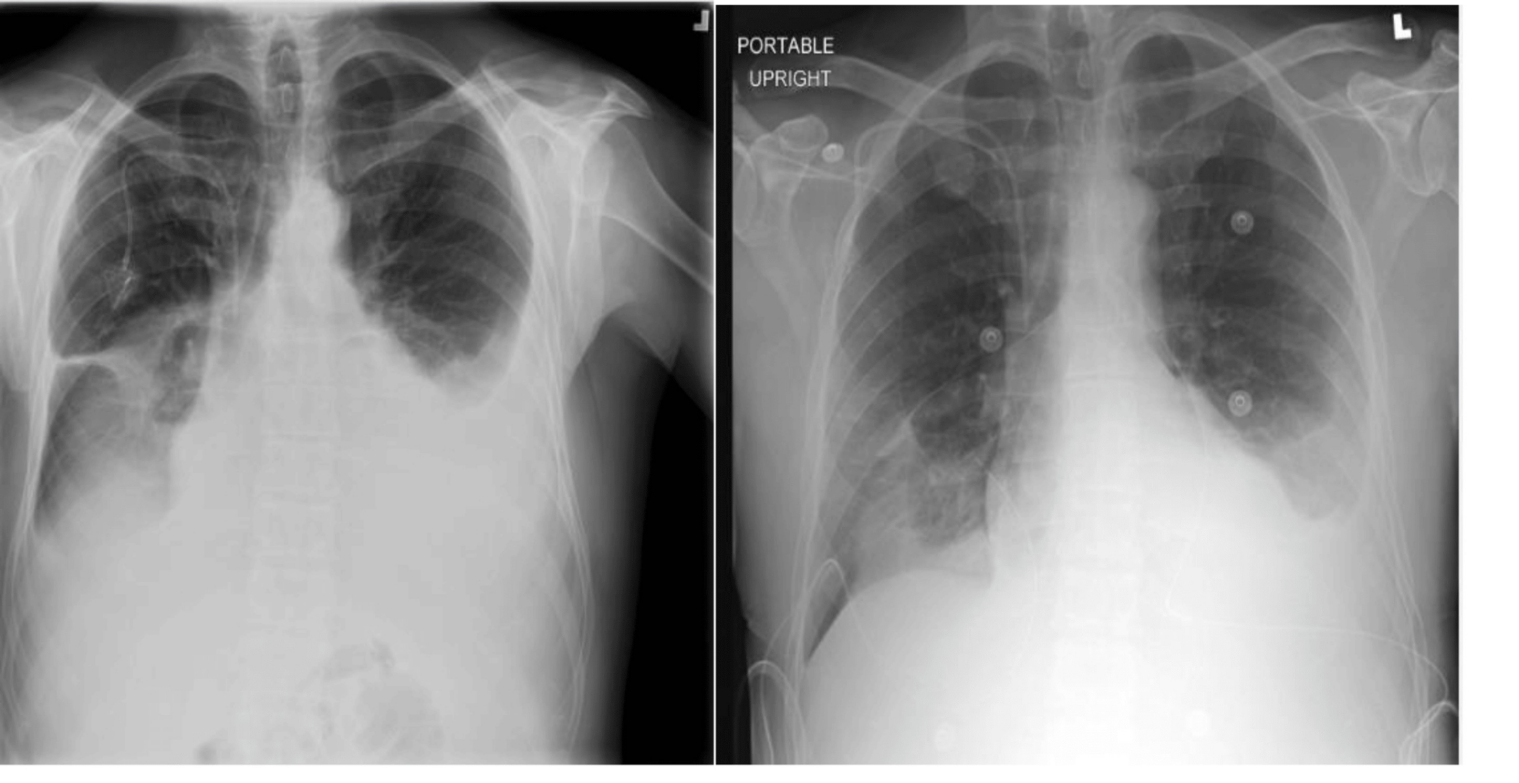
Chest X-ray showing bilateral moderate to large pleural effusions (left) and post indwelling pleural catheter placement with improvement in effusions (right).

A 52-year-old male with a history of relapsed/refractory follicular lymphoma who had progressed through multiple lines of therapy and was recently enrolled in a phase 1 open-label trial of intratumorally administered autologous SIRPα-low macrophages (SIRPant-M) presented to the interventional pulmonary clinic with a history of symptomatic bilateral malignant-related chylous pleural effusions (pleural fluid triglycerides > 1000 mg/dL, protein = 2.7 gm/dl, glucose = 102 mg/dl, and positive cytology for lymphoma involvement). The patient underwent multiple thoracenteses, followed by bilateral indwelling pleural catheter (IPC) placement with significant symptomatic relief. Prior to catheter placement, his blood pressure was 131/89 mmHg, pulse was 104, and he was saturating at 95% on 2 liters of oxygen. His chest X-ray prior to placement of the catheters showed bilateral moderate to large effusions, left > right, and chest X-ray post placement showed improvement in bilateral effusions (Figure 1).

**Figure 2:**
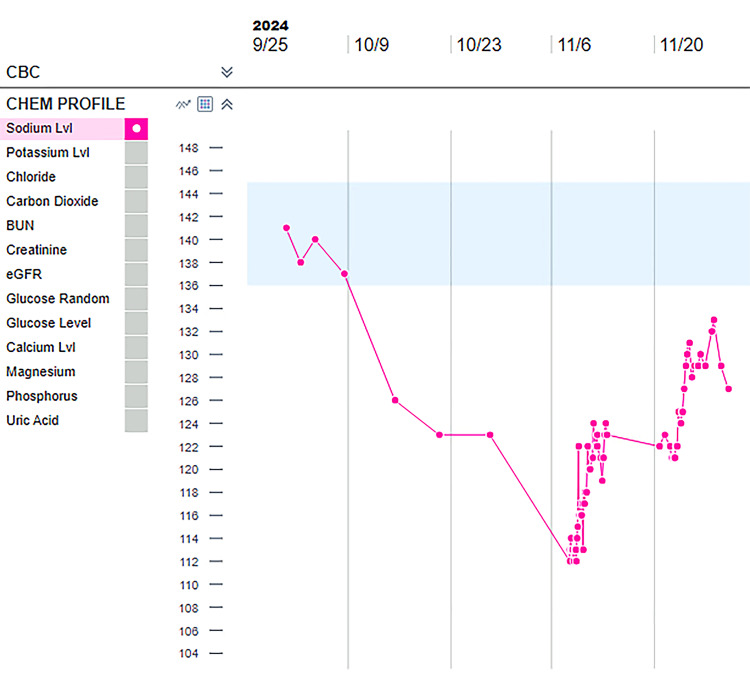
Serum sodium level trend following placement of bilateral indwelling pleural catheters. BUN: blood urea nitrogen; eGFR: estimated glomerular filtration rate.

At a two-week follow-up appointment, he reported a drain output of >1000 mL/day on the left and >750 mL/day on the right. Approximately one month following bilateral IPC placement, the patient was admitted to the intensive care unit (ICU) with severe hypo-osmolar (serum osmolality = 246 mOsm/kg) hyponatremia (sodium = 112 mmol/L) and hyperkalemia (potassium = 6.4 mmol/L), with stable renal function (creatinine = 1.02 mg/dL) and urine osmolality of 565 mOsm/kg. Sodium level on the day of IPC placement was 137 mmol/L, dropped to 126 mmol/L at one week, and has been stable for the subsequent two weeks until his admission (Figure 2). Table 1 depicts the sodium level on the day of IPC placement, one week post IPC placement, two weeks, three weeks, and four weeks post IPC placement.

**Table 1:** Sodium level on the day of IPC placement, one week post IPC placement, two weeks, three weeks, and four weeks post IPC placement. BUN: blood urea nitrogen; IPC: indwelling pleural catheter.

	Reference range & units	Pre-IPC placement	1 week post IPC placement	2 weeks post IPC placement	3 weeks post IPC placement	4 weeks post IPC placement
Sodium	136 - 145 mmol/L	137	126	123	123	112
Potassium	3.4 - 4.5 mmol/L	4.2	5.9	6.0	5.9	6.3
Chloride	98 - 107 mmol/L	104	92	90	92	82
Carbon dioxide	22 - 29 mmol/L	27	25	24	25	20
BUN	6 - 23 mg/dL	28	21	23	21	23
Creatinine	0.67 - 1.17 mg/dL	1.03	1.06	1.13	1.06	1.00

The patient was conscious, oriented, and reported no neurologic symptoms. He was managed with hypertonic saline, free water restriction, and oral urea, and IPC drainage was limited to 500 mL/day bilaterally. He then underwent a lymphangiogram and successful thoracic duct embolization. He was not deemed to be a surgical candidate in view of poor performance status and advanced malignant disease refractory to treatment. He was referred to interventional radiology for a diagnostic lymphangiogram that showed marked narrowing and compression of the thoracic duct in the lower mediastinum and upper abdomen. There were areas suspicious for chyle leakage in the mediastinum, but no definite leak was seen. Given the persistence of the chylothorax and the patient's clinical status, a decision was made to perform the thoracic duct embolization to redirect the chyle flow away from the chest. The patient’s sodium improved to >120 mmol/L with drainage limitation and salt tablets, and he was discharged home with recommendations for ongoing limitation of IPC drainage. He continued to have persistent chylous pleural effusions despite thoracic duct embolization and developed chylous ascites, and ultimately passed away several months later due to disease progression.

## Discussion

Severe hyponatremia following IPC placement in patients with high-output malignancy-related chylous pleural effusions is a rare and infrequently reported complication. To our knowledge, there are no reports of patients experiencing severe hyponatremia in association with IPC placement for malignant chylous pleural effusions, although Andreou et al. reported a case of severe hyponatremia that occurred secondary to a postoperative chylous leak (2650 mL/day) following a neck dissection that had similar clinical features to our case [3]. Our patient presented with severe hypo-osmolar hyponatremia (sodium level of 112 mmol/L), which was attributed to excessive loss of sodium-rich chyle. This is supported by his previously normal sodium level prior to insertion of the bilateral IPCs, and the fact that he was asymptomatic at presentation, which is consistent with a gradual decline in serum sodium that occurred over the weeks following placement. The relatively elevated urine osmolality in the setting of severe hyponatremia observed in this patient may suggest a component of the syndrome of inappropriate antidiuretic hormone secretion (SIADH), and various forms of lymphoma have been associated with the development of SIADH previously [4,5]. Alternatively, the relatively elevated urine osmolality in this case could be explained by hypovolemia alone in the setting of robust IPC drainage, which is what we favor as most likely [6].

The management of high-output malignant chylous pleural effusions is not standardized. Although the use of IPCs is common for the management of malignant pleural effusions, they are less commonly utilized for the management of malignant chylous pleural effusions, although there are reports supporting their use [7,8]. As demonstrated in this patient, high-volume drainage can result in severe hyponatremia with potentially life-threatening consequences. Strategies to avoid this complication may include regular electrolyte monitoring, limiting daily drainage, and early consideration for definitive therapies such as thoracic duct ligation or embolization. The American College of Radiology, in its 2024 Appropriateness Criteria, notes that surgical interventions such as thoracic duct ligation, pleurodesis, or shunt procedures are considered when conservative management fails, but the efficacy in malignancy-related cases is generally lower than in non-malignant etiologies [9]. Most published series include severely ill patients such as ours, and complication rates can be significant, with morbidity up to 38% and mortality up to 25% [10].

## Conclusions

Severe hyponatremia following IPC placement for malignancy-related chylous pleural effusion is a rare and potentially life-threatening complication. Physicians should maintain a high index of suspicion for electrolyte imbalances in high-output malignant chylous pleural effusions after IPC placement and balance the need for drainage for symptomatic relief with the risk for electrolyte derangements. Further research is needed to establish standardized protocols for IPC use and management in this population.
